# Aging of Skeletal Stem Cells

**DOI:** 10.20900/agmr20220006

**Published:** 2022-06-30

**Authors:** M. Gohazrua K. Butler, Thomas H. Ambrosi, Matthew P. Murphy, Charles K. F. Chan

**Affiliations:** 1 Institute for Stem Cell Biology and Regenerative Medicine, Stanford University School of Medicine, Stanford, CA 94305, USA; 2 Blond McIndoe Laboratories, Division of Cell Matrix Biology and Regenerative Medicine, School of Biological Sciences, Faculty of Biology, Medicine and Health, The University of Manchester, Manchester Academic Health Science Centre, Manchester, M13 9PL, UK

**Keywords:** skeletal stem cells, aging, bone, cartilage, regeneration, degeneration

## Abstract

The skeletal system is generated and maintained by its progenitors, skeletal stem cells (SSCs), across the duration of life. Gradual changes associated with aging result in significant differences in functionality of SSCs. Declines in bone and cartilage production, increase of bone marrow adipose tissue, compositional changes of cellular microenvironments, and subsequent deterioration of external and internal structures culminate in the aged and weakened skeleton. The features and mechanisms of skeletal aging, and of its stem and progenitor cells in particular, are topics of recent investigation. The discovery of functionally homogeneous SSC populations with a defined cell surface phenotype has allowed for closer inspection of aging in terms of its effects on transcriptional regulation, cell function, and identity. Here, we review the aspects of SSC aging on both micro- and macroscopic levels. Up-to-date knowledge of SSC biology and aging is presented, and directions for future research and potential therapies are discussed. The realm of SSC-mediated bone aging remains an important component of global health and a necessary facet in our understanding of human aging.

## INTRODUCTION

A remarkable proportion of healthcare costs are attributed to skeletal disease [1]. In 1990, the estimated annual global costs of osteoporosis-related fractures alone was $34 billion and is projected to rise to $131 billion by 2050 as the worldwide elderly population increases [2]. Limits to understanding the mechanics of skeletal diseases have prevented significant progress in developing reliable treatments, thus a better understanding of the molecular and genetic components of aging in skeletal progenitors can provide insight into the key targets for therapeutics in age-related skeletal disease.

Fundamentally, the vertebrate skeleton is a remarkably dynamic and complicated organ. Throughout life, nearly every bone in the body continually deconstructs and regenerates itself in order to maintain structural and mechanical integrity [3]. This highly regulated process allows each bone to acquire and maintain a unique size, density, and shape, constantly repairing and reorganizing its mineral and cellular contents throughout development. In humans, the rate of bone formation exceeds resorption between birth and adulthood; bone mineral density peaks between 20 to 30 years of age, followed by a gradual and terminal decline in mineral density, as well as a decline in the volume of articular cartilage, which does not regenerate [3]. This dynamic generation and resorption of bone is carried out by osteoblasts and osteoclasts respectively, each deriving from separate progenitor populations. The relative population and activity of these cells changes across life according to the needs of the individual and are largely subject to influences such as nutrition, hormones, growth factors, trauma, as well as heritable mutations in genes regulating bone mineral density [4]. Where the lifespan of an individual osteoblast or osteoclast averages between 2 weeks to 3 months, the necessity to replenish these cell populations throughout life is evident [5]. Moreover, the role of healthy bone marrow in housing the progenitors of the skeletal, hematopoietic and immune systems is well understood and further demonstrates the importance of a stable and tightly regulated bone microenvironment [6–8].

## SKELETAL STEM CELLS

Throughout life, adult stem cells are responsible for replenishing and restoring tissues of the body. Evidently the natural process of aging diminishes the capability of adult stem cells to maintain these systems in a youthful and resilient state. In the skeletal system, aging corresponds to a decline in the regenerative and restorative potential of its progenitor cell population, and through replicative exhaustion, chromatin remodeling, and changes to the intra- and extracellular transcriptional landscape, the skeleton becomes less capable of maintaining its dynamic stability [9].

The foundation for skeletal integrity lies in the cellular progenitors of the skeletal system supplying bone tissue with its building blocks of mature cell types. Significant effort has been made in identifying the bona fide progenitors of bone- and cartilage-forming cells, and indeed much debate remains over the precise cellular identity and definition of such progenitors [9,10]. A long-held conclusion that mesenchymal stromal/stem cells (MSCs) give rise to all compartments of the skeletal system has recently been put into question with evidence suggesting MSCs have non-skeletogenic, tissue-specific properties, and consist of highly heterogeneous cell subtypes, including committed lineage progenitors among multipotent progenitors [9,11]. Current protocols for obtaining purified MSCs rely primarily on plastic adherence from treated tissues such as flushed or crushed bone marrow, followed by fluorescent cell sorting sometimes gated by only a single surface marker, however these methods likely select for a population of cells which appear homogeneous in epitope expression but are functional diverse and potentially exclude quiescent stem cells that otherwise do not readily adhere. For example, Nestin was identified as a marker for progenitor populations in bone marrow, however lineage tracing studies have shown that Nestin-expressing MSCs in the marrow and perivascular niches contain lineage-committed osteoblastic and endothelial cells [12,13]. More recently Leptin receptor-expressing MSCs have been described as putative progenitors of the skeletal system, but subsequent functional analyses have shown that very few cells within this population are multipotent or give rise in-vitro to fibroblast colonies [14]. Other markers such as Gremlin1, parathyroid hormone-related protein, and Glioma-associated oncogene 1 have also been used to isolate suspected multipotent skeletal progenitors, although such populations similarly contain a variety of cell types that include lineage-committed cells rather than a homogeneous population of multipotent progenitors [15,16,17].

Only within the past decade have advances in lineage tracing and cell sorting using multiple surface markers revealed more functionally homogeneous populations of self-renewing, lineage-restricted progenitors of bone, cartilage, and stromal cells that have the unique capacity to effectively reconstitute the skeletal niche in-vivo [18–21]. These skeletal stem cells (SSCs), unlike MSCs, are defined by their position at the top of the lineage hierarchy representing functionally homogeneous cell populations that exhibit the necessary properties of a self-renewing and multipotent stem cell [9]. In particular, SSCs demonstrate high clonal capacity and give rise to the cell types involved in maintaining skeletal tissues including bone, cartilage, and stroma, further providing them with the unique ability of generating de novo bone marrow niches at ectopic sites [19,20]. Identification of these highly enriched populations of SSCs rely largely on selecting combinations of cell surface markers that are unique to each population and exclude markers indicative of hematopoietic and endothelial origin. Using comprehensive functional and single-cell transcriptomic analyses of cells from various bone compartments, several bona fide SSC populations have been proposed in regions such as bone marrow, growth plate, periosteum, and perivasculature space, with the potential for each SSC population to possess varying differentiation capacity towards bone, cartilage, stroma, and bone marrow adipose tissue (BMAT) depending on the microenvironment they reside in [19,20,22–25]. This is additionally reflected in the bone forming mechanism by which SSC populations generate skeletal tissues, i.e. intramembranous versus endochondral ossification. Altogether, this indicates unique cellular characteristics between SSCs from separate anatomical subregions and bone compartments [26–28]. Given the recent and dynamic nature of these studies however, the potential for additional refinement to more transcriptionally and functionally homogeneous SSC populations may yet be achieved.

## FEATURES OF AGED SKELETAL STEM CELLS

**Figure 1.**
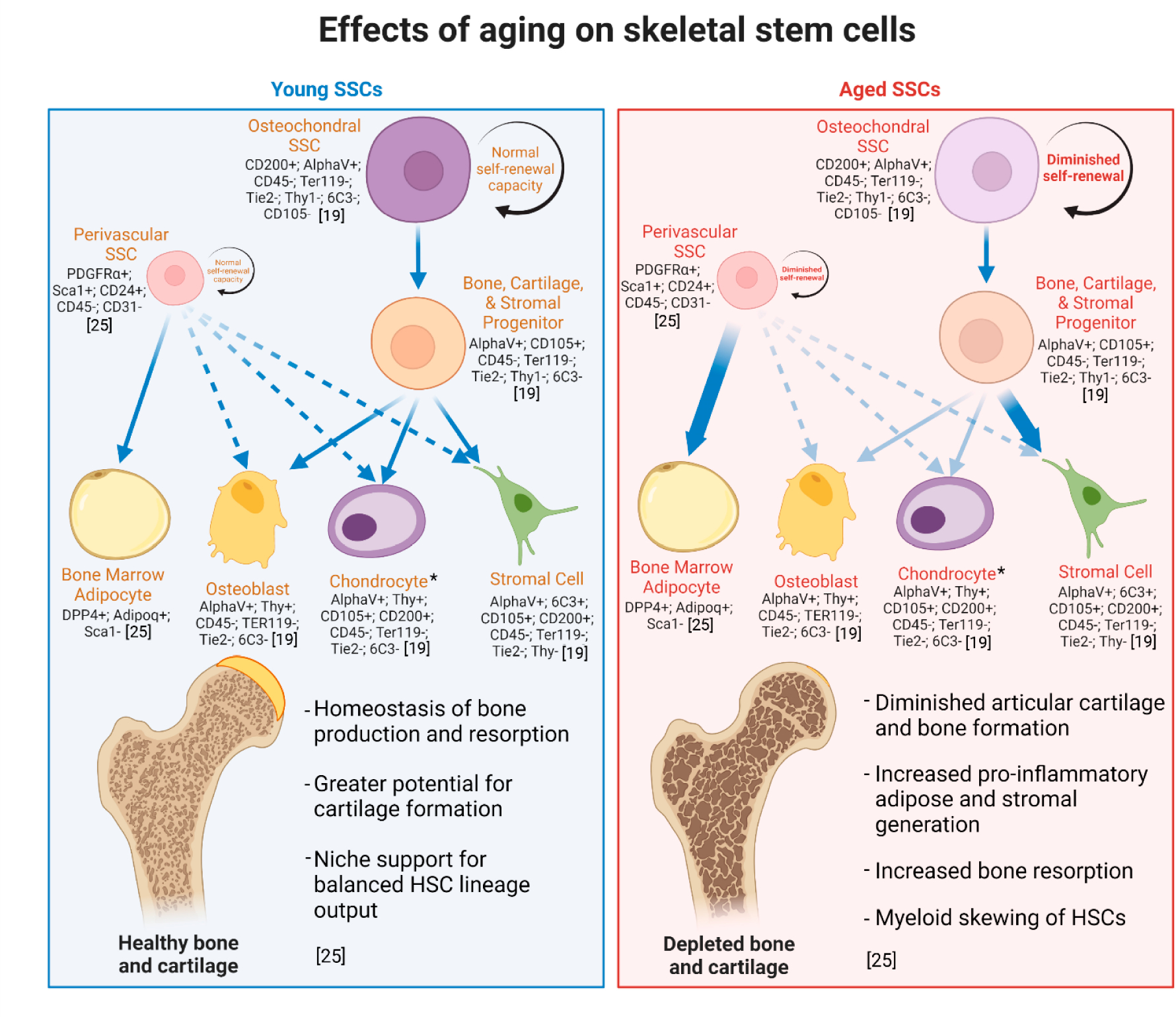
The effects of aging on SSCs. From the bone marrow-derived mouse osteochondral SSCs, the lineage-committed bone, cartilage, and stromal precursor (BCSP) cell gives rise to osteoblasts, chondrocytes, and stromal cells, but not bone marrow adipose. Human perivascular SSCs give rise to bone marrow adipose and under specific circumstances, e.g., fracture—bone, cartilage, and stroma. Aging skews lineage output (indicated by arrows). Surface markers identify skeletal cell types in mice [19,25]. *Chondrocyte generation persists throughout life, however clonal diversity of chondral tissues and generation of articular chondrocytes significantly diminishes with age, while injury-activated SSCs give rise to hypertrophic chondrocytes that generate mostly fibrocartilage [30].

In search of the transcriptomic identity of SSCs, the apparent features of aging in the skeletal system reveal itself (Figure 1). In aged SSCs, downregulation of skeletogenic and cellular senescence-suppressing pathways are associated with diminished capacity for bone formation and ultimately fracture healing [29]. Similarly, the ability for SSCs to differentiate toward articular cartilage decreases with age [29,30]. The lineage potential of aged SSCs is skewed towards stroma, and in aged stromal tissues higher amounts of pro-inflammatory cytokines disrupt maintenance of hematopoietic progenitors in the niche [31]. In the hematopoietic stem cell system, lineage-skewing of myeloid progenitors to the bone-resorptive osteoclasts increases with age, exacerbating the gradual decrease in bone mineral density [31]. A perivascular SSC subtype drives the widely observed increases in BMAT during aging, leading to impairments in regenerative processes of the skeletal system [25,32,33]. Not surprisingly, several studies show bone mineral density is negatively correlated with BMAT, suggesting that aging leads to limitations in the differentiation capacity of SSCs responsible for generating osteochondrogenic cells, ultimately resulting in BMAT accumulation [34,35]. Interestingly, BMAT is higher in men between the ages of 20 to 60 compared to women of the same age group, however this difference is reversed beyond the age of 60 with women having approximately 10% greater fat content than men [36,37]. Postmenopausal women also have greater prevalence of osteoporosis, although the risk for developing osteoporosis increases generally between men and women with age [38,39]. In consequence, the aged skeleton contains both a weakened internal and external structure, resulting in a more substantial likelihood for debilitating bone injury and disease.

## MECHANISMS OF SKELETAL STEM CELL AGING

In many regards, biological aging itself is a process that is poorly understood. Theories on the causes of aging point towards genomic instability and progressively deleterious epigenetic modifications that produces an aged, unstable cellular phenotype [40]. Evidence suggests that telomere attrition strongly correlates with age-related decline in cellular functionality, but can be rescued by artificial lengthening of telomeres through reactivation of telomerase [41,42]. Other sources suggest stem cell exhaustion, cellular senescence, and loss of proteostasis are major contributors to the overall decline in functionality associated with aging, and likely aging involves many of these features in concert [43,44].

The mechanisms of aging in SSCs remain less clear but are under current investigation, with focus on relating the functional differences between young and aged cells. A summary of gene pathways altered in aged cells and their effect on SSCs and skeletal tissues is provided in Table 1. In aged SSCs, skeletogenic WNT signal pathways and negative regulators of cellular senescence are downregulated, while stromal extracellular matrix signaling pathways are upregulated [29]. In this context, aging may increase the number of senescent SSCs in the niche, potentially promoting osteoclastogenesis as demonstrated by in-vitro studies and further weakening the bone architecture [45]. Curiously, aged SSCs show no changes in telomerase activity suggesting that senescence is not necessarily the main driver of bone stem cell aging [31]. In long bones, SSCs lose transcriptomic diversity, fail to activate, and display reduced bone-forming potential during aging [25]. Following microfracture injury, cartilage-derived SSCs at the distal femur of adult mice demonstrate diminished capacity to produce type II collagen indicative of articular cartilage, instead producing MMP13 and types I and X collagen indicative of fibrocartilage and hypertrophic chondrocytes [30]. This may be due in part to diminished signaling from bone morphogenic protein 2 (BMP2) and increased signaling from vascular endothelial growth factor (VEGF), which are known factors in chondrogenic differentiation and induce in-vivo production of articular/hyaline cartilage [19,20,30]. Defects in the production of MMP13 and pro-osteochondrogenic factor TGF-β are known to disrupt cartilage and bone homeostasis, thus aged SSCs themselves give rise to downstream lineages that enhance age-related degeneration of skeletal structures [46,47]. In cells of tissue systems such as vascular endothelial cells, aging may affect the secretion of osteochondrogenic factors in the BMP and WNT pathways and prevent necessary endocrine signaling that orchestrate systemic bone and cartilage remodeling [48,49].

Within the skeletal niche, hematopoietic stem cells (HSCs) reside alongside skeletal cell types and share an age-related decline in lineage diversity potential [53]. A hallmark feature of HSC aging is the decline in the generation of lymphoid progenitors with reciprocal skewing towards myeloid progenitors [53]. The aged skeletal lineage generates elevated levels of pro-inflammatory and pro-osteoclastogenic factors, which contributes to myeloid skewing [31]. For example, increased Colony-stimulating factor-1 (CSF1), a key signaling cue for osteoclastogenesis from SSC-derived cell populations, and its cognate receptor on granulocyte-monocyte progenitors correspond with higher bone-resorbing osteoclast activity during aging, while a decrease in TGF-β results in less osteoblast activity [31]. Furthermore, the greater levels of pro-inflammatory factors in aged cells within the skeletal niche potentially disrupt SSC migration and deplete the healthy niche of HSCs [31,54]. Chronic inflammation, a process that inhibits tissue regeneration in other tissues in the elderly, decreases osteogenic potential of SSCs through age-related activation of NF-κB [50]. In this regard, differences in humoral factors such as increases to pro-inflammatory cytokines, many of which originate in the bone marrow, likely play a critical role in aging of the skeletal system and beyond on the systemic level. Experiments with heterochronic parabiotic mice have demonstrated that circulating systemic cell-extrinsic factors from young blood restore a youthful phenotype to brain, vascular, and muscle cells, however somewhat unexpectedly fails to restore functionality to hematopoietic stem cells [53,55]. Investigation of heterochronic parabiosis in the skeletal system has similarly revealed a failure of young blood to restore osteochondrogenic activity of aged SSCs and bone loss more generally [31]. Thus, the role of systemic humoral factors in the skeletal niche remains to be more clearly elucidated.

Lastly, evidence of age-related differences of SSCs in the histone deacetylation protein Sirtuin1 suggests a mechanism for epigenetic control of SSC differentiation [29]. In this study, selective inhibition of Sirtuin1, resembling the decrease in expression in aged SSCs, decreases osteogenic differentiation, while its activation produced improvement of osteogenesis. In other stem cell systems, Sirtuins regulate proliferation, differentiation, and mitochondrial metabolism, demonstrating epigenetic regulation as a major factor in stem cell function [44,55]. A more in-depth evaluation of the epigenetic and transcriptomic landscape, including the role of alternative splicing and post-translation modification of key pathways in SSC identity, is essential in gaining a clearer understanding of age-related cellular changes in the skeletal system.

## CURRENT AND FUTURE TREATMENTS

Current approaches to treating age-related decline in bone and cartilage health rely almost exclusively on maintaining the balance of bone resorption and production by targeting osteoclast and osteoblast activity [56]. Treatments such as bisphosphonates, NF-κB inhibitors, calcitonin, and selective estrogen receptor modulators decrease bone resorption by inhibiting osteoclastogenesis or mediating osteoclast apoptosis [51,52]. The variety of contraindications and frequency of off-target effects by these treatments, however, reduces the pool of candidate patients and remains a major drawback to treatment of skeletal disease. Similarly, sclerostin inhibitors and parathyroid hormone are used to increase osteoblast activity to promote bone production, but remain limited in efficacy and availability [57,58].

Directed application of stem cell therapies in humans remains largely under investigation, but provides an enticing avenue for minimally disruptive, highly regenerative treatment [59]. Studies in SSCs thus far have demonstrated multiple potential targets for bone and cartilage regeneration; thus, SSC-directed treatment would likely circumvent the limitations to efficacy seen in many clinical trials relying on unspecific MSC-based approaches [9]. Recovery of lost articular cartilage by stimulated SSC differentiation through BMP2 and soluble VEGFR1 has already been demonstrated in mice, and could improve already established surgical models used to treat cartilage loss [30]. Results of another study point towards the prospect of piezoelectrical stimulation to direct SSC migration towards sites of injury with the potential to regenerate hyaline cartilage [60]. Improvement of bone regeneration in aged or injured bone could be achieved through local combinatorial treatment of BMP2 and CSF1 agonist, targeting both SSC and HSC compartments [31]. In one study, upregulation of osteogenic markers RUNX2 and COL1A1 was achieved through mechanical vibrational of in-vitro cultures at 10 MHz frequency over the course of five days, although this remains to be shown in an in-vivo model [61].

Accumulation of senescent SSCs may play a role in age-related skeletal regeneration deficiencies, and indeed in-vivo studies in mice have demonstrated a reversal of age-related bone loss by eliminating senescent cells [45]. Remodeling of stem cell chromatin and transient expression of nuclear reprogramming factors could potentially restore SSCs to a more youthful epigenetic state, increasing the potential for differentiation into osteochondrogenic lineages [62,63]. Less conventional methods, such as growing bone scaffolds from autologous bone marrow within human tissues for transplantation, have also been explored and suggest autologous SSC therapy may be effective in treating both common and rare diseases of the skeletal system [64].

## CONCLUSIONS

In general, our understanding of aging among organisms remains elusive. While the role of aging in nature is not clearly delineated, evidence of unusually long lifespans in the animal kingdom, the longest of potentially 300 or more years belonging to the Greenland shark, suggests the potential for expansion of the human lifespan, and the potential to reverse or elongate youthful features of human tissues through targeting of stem cells [65,66]. Much inspiration for reversal of aging in stem cells is being drawn from examples of developmental reversal in nature, such as in Cnidarians which are known to avoid the deteriorating effects of aging through reverse development and reverting to early tissue progenitor stages [67]. Growing interest in the presence of aging in single celled organisms such as bacteria have revealed ancient mechanisms by which aging may be a fundamental part of cell biology [68]. These studies may propose novel pathways and features of the aging landscape in human stem cells that may otherwise have remained unsuspected.

Indeed, the loom of aging evades no known animal. Regardless, the prospect of delaying or reversing the effects of aging serves as motivation to understand the systems that generate our bodies. Much progress has been made in recent years to reveal the process by which the skeleton is generated and maintained. The discovery of functionally homogeneous SSC populations identified by a combination of surface markers has therefore unveiled the potential for directed and reliable studies into the nature of the progenitor cells of the skeletal system. Moreover, the complexities of skeletal aging can more readily be elucidated by studying the changes in cellular function and expression profile across age groups. Several studies have already identified key features of SSC aging and its components, but more work is needed to better clarify cellular mechanisms that, in aggregate, generate an aged and dysfunctional skeletal niche, and subsequently an aged and dysfunctional skeletal system.

## Figures and Tables

**Table 1.** Regulation of skeletal-related gene pathways and their effects on SSCs and skeletal maintenance.

Gene/Pathway	Regulation in aged cells	Effects on SSCs and the skeletal system
Sirtuin1	Downregulated	Histone deacetylation protein implicated in aging and a target for rejuvenation therapeutics, reactivation of Sirt1 improves osteogenic differentiation in aged SSCs [29].
CSF1	Upregulated	A key signaling cue for osteoclastogenesis from SSC-derived cell populations, increased CSF1 signaling corresponds with higher osteoclast activity, while inhibition promotes osteogenesis of SSCs [5,9,31].
BMP2	Downregulated	Diminished signaling of BMP2 with SSCs may be related to poor formation of bone and articular cartilage; differentiation can be induced through combinatorial treatment with BMP2 and sVEGFR (chondrogenesis) or CSF1 (osteogenesis) [19,20,30,48,49].
VEGF	Upregulated	Increased VEGF signaling contributes to formation of fibrocartilage; combinatorial treatment with BMP2 induces formation of articular cartilage by activated SSCs [19,20,30].
MMP13	Upregulated	Increased production of MMP13 is associated with formation of hypertrophic chondrocytes and fibrocartilage, disrupting homeostasis of articular cartilage and bone [30,46,47].
TGF-β	Downregulated	Diminished TGF-β signaling results in impaired osteoblast activity and decreased bone formation [31,33,46,47].
WNT	Downregulated	Diminished WNT signaling in aged SSCs may contribute to stem cell senescence and promote osteoclast activity [29,48,49].
NF-κB	Upregulated	Increased humoral circulation of NF-κB corresponds with chronic age-related inflammation and diminished osteogenic activity; inhibition of NF-κB in aged cells restores youthful phenotype [50–52].
